# Evaluation and Design of Genome-Wide CRISPR/SpCas9 Knockout Screens

**DOI:** 10.1534/g3.117.041277

**Published:** 2017-06-26

**Authors:** Traver Hart, Amy Hin Yan Tong, Katie Chan, Jolanda Van Leeuwen, Ashwin Seetharaman, Michael Aregger, Megha Chandrashekhar, Nicole Hustedt, Sahil Seth, Avery Noonan, Andrea Habsid, Olga Sizova, Lyudmila Nedyalkova, Ryan Climie, Leanne Tworzyanski, Keith Lawson, Maria Augusta Sartori, Sabriyeh Alibeh, David Tieu, Sanna Masud, Patricia Mero, Alexander Weiss, Kevin R. Brown, Matej Usaj, Maximilian Billmann, Mahfuzur Rahman, Michael Costanzo, Chad L. Myers, Brenda J. Andrews, Charles Boone, Daniel Durocher, Jason Moffat

**Affiliations:** * Department of Bioinformatics and Computational Biology, The University of Texas MD Anderson Cancer Center, Houston, Texas 77030; † Donnelly Centre, University of Toronto, Ontario M5S3E1, Canada; ‡ The Lunenfeld-Tanenbaum Research Institute, Mount Sinai Hospital, Toronto, Ontario M5G1X5, Canada; § Department of Genomic Medicine, The University of Texas MD Anderson Cancer Center, Houston, Texas 77030; ** Department of Molecular Genetics, University of Toronto, Ontario M5S3E1, Canada; †† Department of Computer Science and Engineering, University of Minnesota, Minneapolis, Minnesota 55455; ‡‡ Canadian Institute for Advanced Research, Toronto, Ontario M5G1Z8, Canada

**Keywords:** genetic screens, CRISPR/Cas9, core essential genes, cancer cell lines

## Abstract

The adaptation of CRISPR/SpCas9 technology to mammalian cell lines is transforming the study of human functional genomics. Pooled libraries of CRISPR guide RNAs (gRNAs) targeting human protein-coding genes and encoded in viral vectors have been used to systematically create gene knockouts in a variety of human cancer and immortalized cell lines, in an effort to identify whether these knockouts cause cellular fitness defects. Previous work has shown that CRISPR screens are more sensitive and specific than pooled-library shRNA screens in similar assays, but currently there exists significant variability across CRISPR library designs and experimental protocols. In this study, we reanalyze 17 genome-scale knockout screens in human cell lines from three research groups, using three different genome-scale gRNA libraries. Using the Bayesian Analysis of Gene Essentiality algorithm to identify essential genes, we refine and expand our previously defined set of human core essential genes from 360 to 684 genes. We use this expanded set of reference core essential genes, CEG2, plus empirical data from six CRISPR knockout screens to guide the design of a sequence-optimized gRNA library, the Toronto KnockOut version 3.0 (TKOv3) library. We then demonstrate the high effectiveness of the library relative to reference sets of essential and nonessential genes, as well as other screens using similar approaches. The optimized TKOv3 library, combined with the CEG2 reference set, provide an efficient, highly optimized platform for performing and assessing gene knockout screens in human cell lines.

The generation of gene knockouts is a cornerstone of functional genomics research. The application of CRISPR technology to induce targeted DNA double-strand breaks in mammalian cells (Jinek *et al.* 2012), coupled with the ability of the endogenous cellular DNA repair machinery to introduce indels when repairing these lesions, has led to the rapid development of pooled-library CRISPR knockout screens in mammalian cells for functional genomics, chemogenomics, the identification of cancer cell vulnerabilities, and other applications (Hart *et al.* 2015; Koike-Yusa *et al.* 2014; Shalem *et al.* 2014; Wang *et al.* 2014, 2015, 2017; Parnas *et al.* 2015; Tzelepis *et al.* 2016; Aguirre *et al.* 2016).

CRISPR screens represent a major advance over pooled-library shRNA screens (Evers *et al.* 2016), the prior state-of-the-art in mammalian functional screening approaches, in both sensitivity and specificity. The current designs of large-scale CRISPR experiments benefited from the many lessons learned in shRNA screening. In particular, the design of early CRISPR libraries to include several guide RNAs (gRNAs) targeting each gene has been driven by experience with pooled-library shRNA screens (Kaelin, 2012; Echeverri *et al.* 2006), as well as the unknowns surrounding the application of CRISPR technology in human cells on a large scale. Integrated analysis of multiple reagents targeting the same gene should overcome the noise introduced by variable reagent effectiveness and the unknown frequency and impact of off-target effects.

With several panels of whole-genome cell-line screens published (Aguirre *et al.* 2016; Hart *et al.* 2015; Tzelepis *et al.* 2016; Wang *et al.* 2015, 2017), the opportunity now exists to undertake a meta-analysis as a means to uncover the drivers of screen quality and variability. Thus, we reanalyzed sets of CRISPR screens conducted in adherent and suspension cell lines, using three different large-scale libraries, and evaluated each for quality and consistency. Based on these observations, we refined our list of core essential genes (CEG), *i.e.*, the set of genes that are likely to be essential in all human cell lines. We evaluated the impact of experimental design, including library size, number of replicates, and use of nontargeting controls, on screen performance. Finally, we derived a sequence signature for highly effective gRNAs and designed an optimized, genome-scale CRISPR library for efficient screening of human cell lines.

## Materials and Methods

The supplemental file HART_data_and_python_notebooks.tgz is available at http://tko.ccbr.utoronto.ca/ for download, and contains python notebooks and all required data to generate the figures presented here. As such, it contains a near-complete, granular description of the computational methods applied in this study. Detailed experimental methods and a summary of computational methods are included below.

### Analysis of screens from various libraries

gRNA readcount data were downloaded from Hart *et al.* (2015), Koike-Yusa *et al.* (2014), Tzelepis *et al.* (2016), and Wang *et al.* (2015). Fold-changes were calculated by normalizing each sample to 10 million reads and calculating log2 (experimental/control) for each replicate of each sample at each timepoint. Control was either the gRNA counts from the genomic DNA collected after infection (TO) or library plasmid pool, depending on the experimental design. A pseudocount of 0.5 reads was added to all readcounts to prevent discontinuities from zeros. gRNA with <30 reads at the T0 timepoint were excluded from the fold-change calculation.

Fold-changes were processed with the Bayesian Analysis of Gene Essentiality (BAGEL) algorithm (Hart and Moffat 2016), using the essential and nonessential training sets defined in Hart *et al.* (2014). The resulting Bayes Factors (BFs) for all screens are included in Supplemental Material, Table S1.

After the Core Essential Genes 2.0 (CEG2) set was defined, BFs for all screens were recalculated using this new reference set (Table S2).

### Identification of CEG2

Of the 17 gRNA screens initially evaluated, three were withheld for later evaluation and analysis. Two others were excluded for relatively poor performance. For the remaining 12 screens, the BF and the number of gRNAs targeting the gene were considered. Note that the number of gRNAs may vary by cell line and by library since only gRNAs with >30 reads in the T0 control sample were used for each cell line screen.

A gene was defined as effectively assayed if it was targeted by at least three gRNAs in a given screen. The CEG2 set was defined as genes effectively assayed in at least seven cell lines, with a strict BF threshold of ≥6 in 85% of cell lines in which they were assayed. Since most genes were assayed in either seven or 12 cell lines, this effectively means that core essentials are hits in six of seven or 11 of 12 screens.

The strict BF ≥ 6 threshold corresponds to a posterior probability of ∼90%. To calculate posterior odds from a BF, it is necessary to multiply by a ratio of priors. Based on significant empirical screening evidence, we estimate ∼10% of genes to be essential in a given cell line; the prior ratio P(essential)/P(nonessential) is therefore 0.1/0.9, which in log2 is ∼−3. Therefore, a BF of three corresponds to a posterior log odds of ∼0, or posterior probability of essentiality of 50%, the threshold we generally apply for identifying essential genes (provided a FDR threshold is also met). A BF of six therefore has posterior log odds of three, or posterior probability of ∼90%.

### Evaluation of gRNA per gene

The Sabatini library in Wang *et al.* (2015) contains 10 gRNA per gene. For each of the five screens in four cell lines (KBM7 is screened twice), a subset of guides was randomly selected and BFs for all genes were calculated from the subset. This process was repeated 10 times for each count of *k* = 2 to *k* = 7 gRNA per gene. Performance for each iteration was evaluated by counting the fraction of core essentials recovered and the overall number of hits called at a defined threshold (BF > 3, FDR < 5%). The mean and SD (*n* = 10 replicates) at each *k* were calculated and plotted in Figure 2.

### Evaluation of replicates per screen

The Toronto KnockOut version 1.0 (TKOv1) screen in RPE1 cells (Hart *et al.* 2015) and Yusa library screen in HL60 cells (Tzelepis *et al.* 2016) were conducted with similar three-replicate experimental designs. We ran BAGEL on each replicate independently, and on all three combinations of two replicates, and evaluated performance of each as for gRNA per gene.

### Nontargeting *vs.* nonessential controls

The Sabatini library contains ∼1000 nontargeting gRNA controls (Wang *et al.* 2015). We compared the distribution of fold-changes for gRNA nontargeting controls to the distribution of fold-changes for gRNA targeting gold-standard nonessential genes. Statistical significance was calculated by *T*-test.

### Identifying sequence signatures

To identify a sequence signature that predicts high-performing guides, we evaluated data from TKOv1 screens. From the base 90k TKOv1 library (Hart *et al.* 2015), we identified genes in the new CEG2 set that were targeted by six gRNAs each. gRNAs were ranked by log fold-change, and the three gRNAs with the best (most negative) fold-change were identified, as well as the worst (remaining three gRNA). Then, the frequency of each nucleotide at each position in the 20-mer guide sequence was calculated for all best guides targeting all selected genes, and the same was done for the worst guides. The worst frequency was subtracted from the best, resulting in a Δ-frequency table. This process was repeated independently for each replicate at the endpoint for six TKOv1 90k library screens (DLD1, GBM, HAP1, HCT116, RPE1, and RPE1dTP53) for a total of 16 samples.

The Δ-frequency tables were summed across the 16 samples and scaled so that the most extreme value (C18) equals one. As TKOv1 explicitly excludes gRNA with T in the last four positions, no score is discovered here; we manually set the score to −1 at these four positions. The final score table is in Table S4. To calculate the sequence score of any candidate gRNA sequence, simply add the nucleotide scores at each position of the gRNA.

The score table was evaluated against the 85k supplementary TKOv1 library, which was only applied to HCT116 and HeLa. We calculated the sequence score for all gRNA targeting essential genes, then compared the fold-change distribution of gRNA in the top quartile of scores to the gRNA in the bottom quartile. We repeated this process for the Yusa, Sabatini, and GeCKO v2 libraries.

### Toronto KnockOut version 3 library design

Gene models for protein-coding genes were derived from Gencode v19 gene models (all genomic analysis was done with hg19). Coding exons were numbered in ascending order from the transcription start site.

The genome was scanned and a list of all candidate gRNA targeting all genes was generated, for all candidate gRNA such that the SpCas9 cut site would be in a coding region. Candidate gRNAs were then filtered for the following criteria: 40–75% GC content, no homopolymers of length of four or greater, no restriction sites for *Age*I (ACCGGT), *Kpn*I (GGTACC), *Bve*I/*Bsp*MI (ACCTGC), *Bsm*I (GAATGC), or *Bsm*BI (CGTCTC), and no common SNP (db138) in either the PAM or the protospacer sequence.

Remaining gRNAs were mapped to hg19 with Bowtie (with NGG PAM sequence appended), allowing up to two mismatches outside the PAM “n” position. Guide sequences were excluded if there was any match within either exonic or intronic sequences of an off-target gene. Guide sequences were then assigned to class 1 in the very rare case where the sequence matches the target gene more than once and does not have any predicted off-target cut sites. Remaining guides were assigned as class 2 (hits targeted gene once, no off-targets; common), class 3 (up to two off-target hits within two mismatches, if off-target sites are in intergenic regions; very common), or class 4 (up to three off-target intergenic hits). The sequence score was also calculated for each guide, with the median sequence score across all guides being ∼−1. In general, a small number of intergenic off-target cut sites has been shown to have negligible impact on cellular fitness relative to guides targeting known nonessential genes (Hart *et al.* 2015; Koike-Yusa *et al.* 2014). The Toronto KnockOut version 3 (TKOv3) library exploits this observation by allowing one to two predicted intergenic off-target cut sites if the sequence score of the primary target site is particularly high. Guides were further binned into ranks, as shown in Table 1.

**Table 1:** Number of guide RNAs ranked by sequence score (SeqScore) and included in the TKOv3

Rank	Class	SeqScore	No. of Candidate Guides	No. of gRNAs Added	Cumulative gRNAs in Library
1	1	>0.0	1501	679	679
2	1,2,3	>0.85	286,415	63,834	64,513
3	1,2,3,4	>0.85	307,059	1069	65,582
4	1,2,3	0.0–0.85	304,477	3476	69,058
5	1,2,3,4	−1.0–0.85	837,136	1890	70,948

To select guides for the library, we began with the rank 1 candidate gRNAs. Then for each exon, we selected the single candidate gRNA with the top sequence score and added it to the library. This process was repeated four times, to allow up to four gRNAs per gene, with the competing goals of maximizing gRNA quality and exon coverage while allowing multiple gRNAs per exon if the gRNAs are high quality. The process therefore selects high-scoring guide sequences for four different exons, if available. If not, high-scoring guides targeting already-targeted exons are prioritized above low-scoring guides targeting different exons. These steps were repeated for each subsequent rank, with the results shown in Table 1. An additional 142 control sequences targeting EGFP, LacZ, and luciferase were also added, for a final library size of 71,090 gRNAs.

### Genome-scale lentiviral gRNA construction

All 71,090 gRNAs were synthesized as 58-mer oligonucleotides on one microarray chip (Custom Array) and amplified by PCR as a pool. The PCR products were purified using QIAquick nucleotide removal kit (Qiagen) and cloned into a modified version of the all-in-one lentiviral vector lentiCRISPRv2 (Addgene), which contains the fSpCas9 gene. The lentiCRISPRv2 vector was digested with FastDigest Esp3I (Thermo Fisher Scientific) and treated with shrimp alkaline phosphatase (NEB) for 30 min at 37°, heat-inactivated for 10 min at 65°, and gel-purified using the QIAquick Gel Extraction kit (Qiagen). Using a one-step digestion and ligation reaction, purified library PCR pool was cloned into the digested lentiCRIPSRv2 vector at a ratio of 1:5 vector-to-insert molar ratio. The ligation reaction was precipitated using Pellet Paint Co-Precipitant (EMD Millipore) and 1 µl of the precipitated ligation was transformed into Endura ElectroCompetent cells (Lucigen). To yield a 1200-fold representation of the library, 10 identical ligation reactions were pooled and purified followed by 40 parallel transformations. Outgrowth media from transformations were pooled and plated onto 100 15-cm LB-carbenicillin (100 µg/ml) plates. Colonies were scraped off plates, pooled, and the plasmid DNA was extracted using the QIAfilter Plasmid Mega kit (Qiagen).

### Cell culture

HEK293T cells were maintained in Dulbecco’s modified Eagle’s medium (DMEM) with high glucose and pyruvate supplemented with 10% FBS and 1% penicillin/streptomycin (Thermo Fisher Scientific). HAP1 cells were obtained from Horizon Discovery and maintained in Iscove’s Modified Dulbecco’s Medium supplemented with 10% FBS and 1% penicillin/streptomycin. All cells were maintained in humidified incubators at 37° and 5% CO_2_.

### Lentivirus production

TKOv3 library lentivirus was produced by cotransfection of lentiviral vectors psPAX2 (packaging vector) and pMDG.2 (envelope vector) with TKOv3 lentiCRISPR plasmid library, using X-tremeGene 9 transfection reagent (Roche). Briefly, HEK293T cells were seeded at a density of 9 × 10^6^ cells per 15-cm plate and incubated overnight, after which cells were transfected with a mixture of psPAX2 (4.8 µg), pMDG.2 (3.2 µg), TKOv3 plasmid library (8 µg), and X-tremeGene 9 (48 µl), in accordance with the manufacturer’s protocol. At 24 hr after transfection, the medium was changed to serum-free, high BSA growth medium (DMEM, 1% BSA, 1% penicillin/streptomycin). Virus-containing medium was harvested 48 hr after transfection, centrifuged at 1500 rpm for 5 min, and stored at −80°. Functional titers in HAP1 cells were determined by infecting cells with a titration of TKOv3 lentiviral library in the presence of polybrene (8 µg/ml). At 24 hr after infection, medium was replaced with puromycin (2 µg/ml) containing medium to select for transduced cells, and incubated for 48 hr. The multiplicity of infection (MOI) of the titrated virus was determined at 72-hr after infection by comparing the percent survival of infected cells to noninfected control cells.

### Pooled genome-wide CRISPR dropout screens in HAP1 cells

A total of 50 × 10^6^ HAP1 cells were infected with TKOv3 lentiviral library (71,090 gRNAs) at an MOI of ∼0.3 to achieve ∼200-fold coverage of the library after selection. At 72 hr after infection, selected cells were split into three replicates containing 15 × 10^6^ cells each, passaged every 3–4 d, and maintained at 200-fold coverage. A total of 15 × 10^6^ cells were collected for genomic DNA extraction at day 0 and at every passage until day 18 after selection, or ∼15 doublings.

Genomic DNA was extracted from cell pellets using the QIAamp Blood Maxi Kit (Qiagen), precipitated using ethanol and sodium chloride and resuspended in EB buffer. gRNA inserts were amplified via PCR using primers harboring Illumina TruSeq adapters i5 and i7 barcodes, and the resulting libraries were sequenced on an Illumina HiSeq2500. Each read was completed with standard primers for dual indexing with Rapid Run V1 reagents. The first 20 cycles of sequencing were dark cycles, or base additions without imaging. The actual 26-bp read begins after the dark cycles and contains two index reads, reading the i7 first, followed by i5 sequences.

### Data availability

The TKOv3 library is available upon request, and for research use from Addgene. All data and supporting information is available at http://tko.ccbr.utoronto.ca/ for download.

## Results

### An updated set of gold-standard CEGs: CEG2

**Figure 1:**
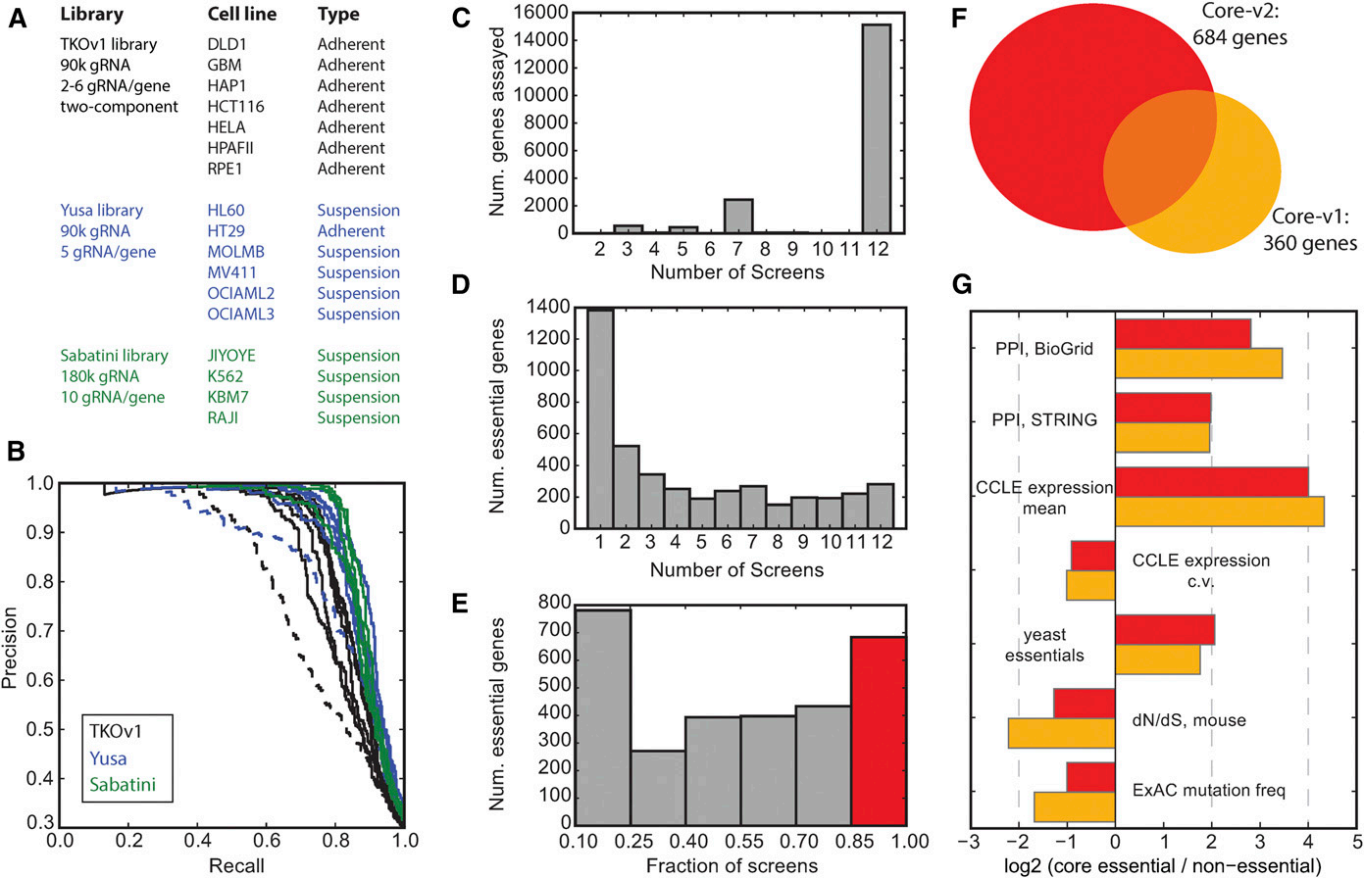
(A) List of CRISPR knockout screens used for this study. (B) Precision-recall curves for the screens in (A) using gold standards defined in Hart *et al.*, 2014. Dashed lines represent low-performing screens that were excluded from further analysis. (C) Number of genes assayed by at least three gRNA per gene, across the 12 screens. (D) Number of genes classified as essential (BF ≥ 6, FDR ≤ 3%) across the 12 screens. (E) Fraction of screens in which a gene is classified as essential. Genes assayed in at least seven screens and essential in 85% of screens (red) are CEG2. (F) CEG2 (*n* = 684) is substantially larger and only overlaps CEG1 (*n* = 360; Hart *et al.* 2014) by ∼50%. (G) Functional characterization of CEG2 (Core-v2) *vs.* CEG1 (Core-v1).

We applied our BAGEL analysis pipeline (Hart and Moffat 2016) to panels of pooled-library CRISPR dropout screens from three different groups, each of which used their own custom library (Hart *et al.* 2015; Koike-Yusa *et al.* 2014; Tzelepis *et al.* 2016; Wang *et al.* 2015) (Table S1). Additional genome-scale pooled screens were recently published, but those were not included in this initial analysis (Aguirre *et al.* 2016; Wang *et al.* 2017). Using the previously published reference sets of essential and nonessential genes (Hart *et al.* 2014), we classified essential and nonessential genes from seven adherent cell lines screened with the TKOv1 library (Hart *et al.* 2015; Steinhart *et al.* 2017), four suspension cell lines using the Sabatini library (Wang *et al.* 2015), and five suspension plus one adherent line screened with the Yusa library (Koike-Yusa *et al.* 2014; Tzelepis *et al.* 2016), for a total of 17 whole-genome screens (Figure 1A and Table S1). Although the screens were performed with different libraries and carried out in different laboratories, the experimental designs are largely similar, with each screen involving a pooled lentiviral library infection of a large number of cells, serial passaging over 2–3 wk, PCR amplification of gRNA integration events, and comparison of the relative abundance of endpoint gRNAs to those of a control timepoint collected shortly after infection.

We removed two screens for relatively poor performance (HeLa and MV411) and withheld an additional three for validation studies (HT29, RPE1, and K562) (Figure 1B). With the remaining 12 high-performing screens (including five adherent and seven suspension cell lines), we defined an updated set of CEGs. We defined a gene as being effectively assayed in a cell line if it was targeted by at least three independent gRNAs; most genes were assayed in all 12 screens, based on library representation (Figure 1C). Genes that were assayed in at least seven out of 12 cell lines and were classified as essential (BF ≥ 6 at FDR ≤ 3%; Figure 1D) in all, or all but one of them (maximum of one putative false negative allowed), were defined as CEG2 (Figure 1E and Table S2).

Compared with our previously defined CEG1 set, which were derived from a panel of pooled-library shRNA screens (Hart *et al.* 2014), CEG2 is 90% larger (*n* = 684 for CEG2 *vs.* *n* = 360 for CEG1; Figure 1F), largely due to the increased sensitivity of CRISPR screens in identifying essential genes at moderate expression levels (Hart *et al.* 2015). However, the CRISPR-derived CEG2 set only includes half of the shRNA-derived CEG1 set (183 of 360). We expect that some of the shRNA-specific hits are true essential genes; for example, >20 genes coding for ribosomal subunits are included in this list. These genes are not well assayed in most CRISPR libraries due to the difficulty in identifying unique gRNA sequences with low probability of off-target cleavage. However, 131 of the 177 shRNA-only genes are assayed in all 12 CRISPR screens; of these, 26 (20%) were never classified as essential in any CRISPR screen and an additional 24 (18%) were scored as essential in one to three CRISPR screens. This suggests that the shRNA-only genes may contain a significant number of false positives, possibly resulting from off-target effects on other essential genes. In contrast, the 501 CRISPR-only additions to the CEG2 set are highly conserved, constitutively expressed, and are central in protein–protein interaction networks (Figure 1G).

**Figure 2:**
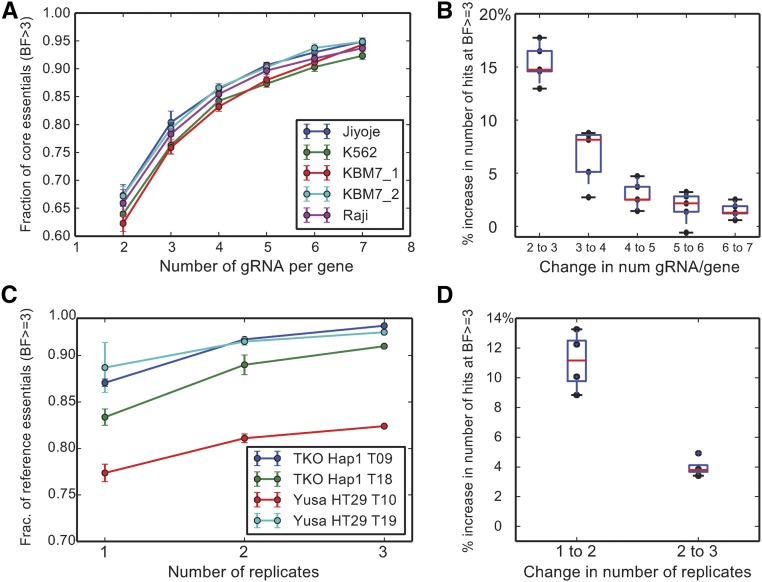
Effect of experimental design on screen performance. (A and B) Effect of number of gRNA per gene. (A) Subsets of the Sabatini library were randomly selected and evaluated using BAGEL. The fraction of CEG2 detected is plotted as a function of the number of gRNA per gene. Error bars represent SD of 10 random samples from the Sabatini library. (B) Incremental increase in the total number of essential genes per screen *vs.* incremental increase in the number of gRNA per gene. (C and D) Effect of number of replicates per experiment. The TKOv1 screen in HAP1 cells and the Yusa screen in HT29 cells, each screened at multiple timepoints, were reanalyzed using all combinations of one, two, or three replicates per screen. (C) The fraction of CEG2 reference essentials identified *vs.* the number of replicates. (D) The incremental increase in total number of essential genes as the number of replicates is increased.

With a strict set of genes included in CEG2, we explored how varying experimental design affected the sensitivity of genome-scale CRISPR screens. We determined the minimum number of gRNAs per gene necessary for a high-quality screen, using the CEG2 as positive controls, after recalculating BFs for all screens using the new training set (Table S3). Using data from the Sabatini screens, which used a library of 10 gRNAs per target gene, we randomly selected subsets of two to seven gRNAs per gene and reran the screens *in silico*. At a fixed BF threshold, the fraction of core essentials (Figure 2A) and total number of hits increased continuously as the number of gRNAs per gene was increased, although at a diminishing rate (Figure 2B). Most of the increased performance was gained with four gRNAs per gene; additional gRNAs per gene added <5% more hits per gRNA added to the screens.

We also considered the number of replicates for each experiment. Using TKOv1 data from a screen of HAP1 cells and Yusa data from HT29 colorectal cancer cells, which were each performed with three replicates, we measured the performance of one, two (all combinations), or three replicates on screen performance. As expected, additional replicates consistently increased the fraction of core essentials (Figure 2C) and the total number of genes called as hits in each screen, but again with diminishing returns: the second replicate increased the number of hits by 9–14%, while the third added <5% (Figure 2D). These results indicate that there are rapidly diminishing returns for additional gRNAs per gene and more than two replicates.

**Figure 3:**
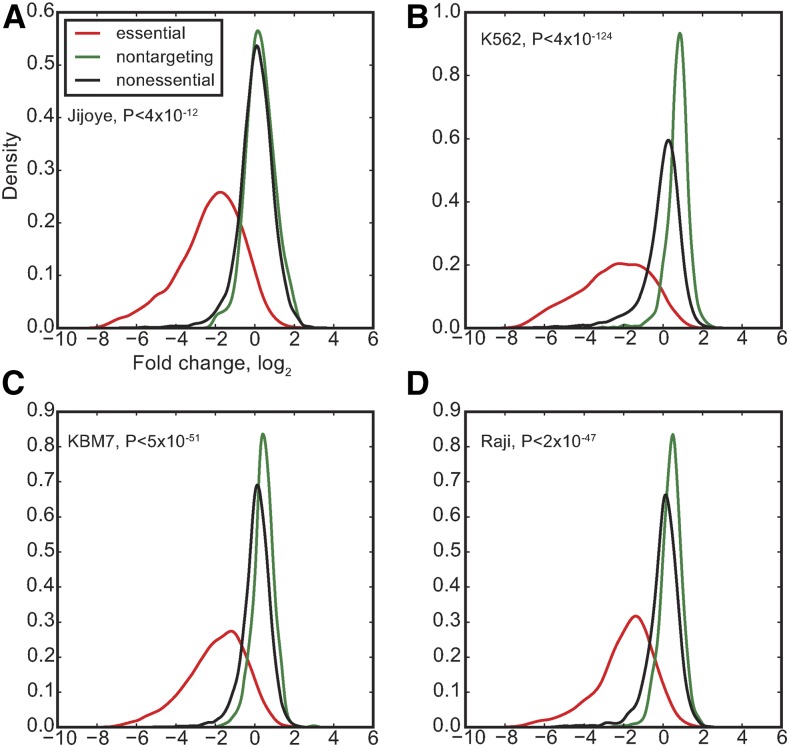
Nonessentials *vs.* nontargeting controls. The distribution of observed fold-changes of gRNA targeting nonessential genes (black) is compared to the distribution for nontargeting control gRNA (green), in the Sabatini screens of Jiyoye (A), K562 (B), KBM7(C), and Raji (D) cells; P-value from *T*-test. For reference, the fold-change of gRNA targeting essential genes is also shown (red).

Finally, we examined the use of nontargeting controls *vs.* controls that target known or suspected nonessential genes. We identified 1014 nontargeting control guides in the Sabatini library and compared their performance with control guides targeting nonessential genes that we have previously defined (Hart *et al.* 2014). To our surprise, nontargeting controls showed significantly different fold-change distributions than those of guides targeting nonessential genes (Figure 3, A–D). Since fold-change is calculated by normalizing read counts and then comparing frequencies, the largest population of minor-phenotype gRNAs will have calculated fold-changes of ∼0. With approximately eightfold more gRNAs targeting nonessential genes than nontargeting controls, the larger population has a fold-change distribution centered at ∼0, while the smaller population appears to have a positive fold-change. In truth, the nontargeting controls likely reflect wild-type growth, while the nonessential controls reflect some small fitness defect from SpCas9-induced cleavage and double-strand break repair, without any locus-specific phenotype. Guides targeting genes with a knockout fitness phenotype will have some combination of locus-specific and nonspecific fitness defect. Locus-specific effects might include the toxicity induced by multiple double-strand breaks in highly amplified regions (Aguirre *et al.* 2016; Munoz *et al.* 2016), but this effect should be mitigated by using a large set of negative control genes that are well-distributed across the genome. Overall, when globally screening for gene-specific fitness effects, it appears that gRNAs inducing DNA double-strand breaks without a gene-specific growth phenotype are a more robust negative control set than nontargeting gRNAs.

### Designing an optimized library

**Figure 4:**
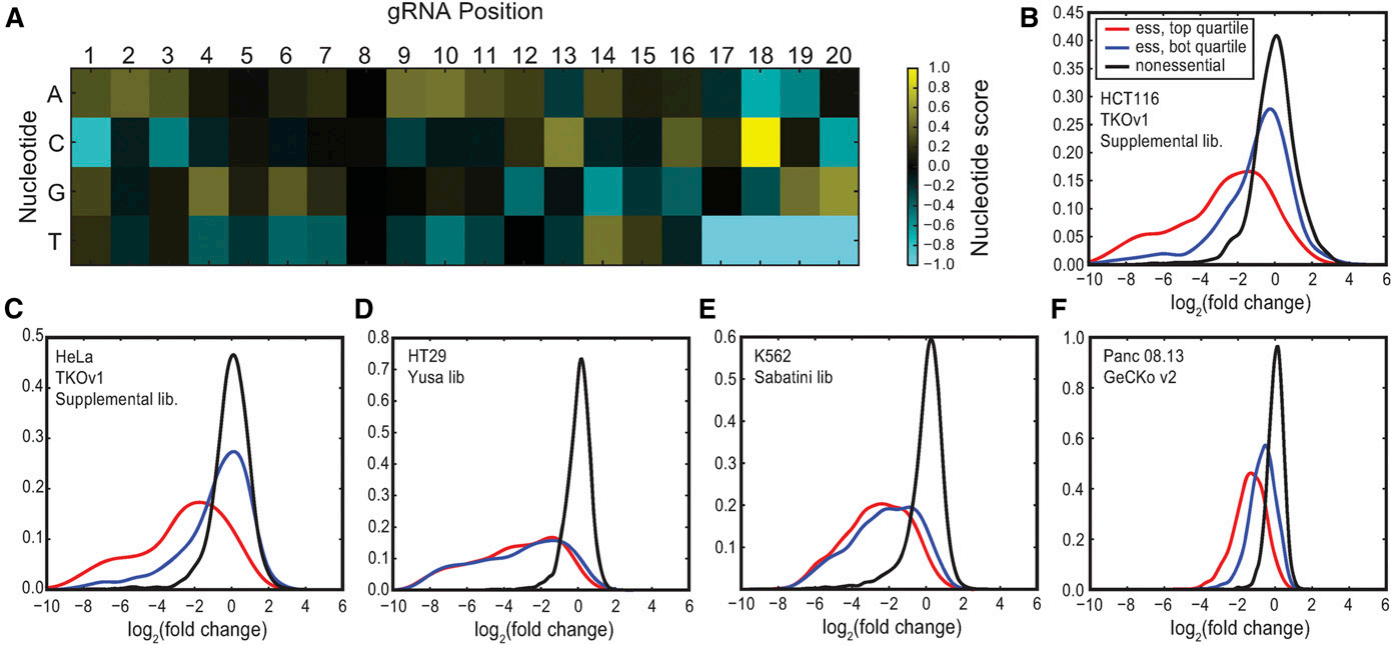
Sequence signature of high-performing guides. (A) Heatmap of the guide score derived from high-performing guides in TKOv1 screens. (B) Across the TKOv1 supplemental library in the HCT116 screen, gRNA targeting CEG2 with sequence scores in the top quartile (red) are compared with gRNA with scores in the bottom quartile (blue), and guides targeting nonessential genes are shown in black. (C–F) Similar plots for TKOv1 (HeLa screen), Yusa, Sabatini, and GeCKOv2 libraries.

Given a set of expected outcomes, *i.e.*, that all CEGs should drop out of a population in a pooled-library screen, we sought to design a sequence-optimized gRNA library that takes advantage of the experimental design characteristics outlined above. Though a variety of small- and medium-scale experiments have been used to guide gRNA selection algorithms (Doench *et al.* 2016; Haeussler *et al.* 2016; Heigwer *et al.* 2014, 2016; Hsu *et al.* 2013; Liu *et al.* 2016; Park *et al.* 2016), to our knowledge our design is based on the largest available set of empirical screening results. Using endpoint data from six TKOv1 screens (DLD1, GBM, HAP1, HCT116, RPE1, plus RPE1dTP53, an RPE1-derived cell line), we selected CEGs targeted by six gRNAs that are each represented by at least 30 reads in the T0 control sample (*n* = 263–360 genes). We rank-ordered the gRNAs for each gene by fold-change and separated the top three (best) and bottom three (worst) into separate lists (numbering 789–1077 gRNA each). We calculated the nucleotide frequency at each position among the best and worst guides across all the screens, subtracted the worst from the best, and normalized the table such that the maximum score at a nucleotide position = 1 (see *Materials and Methods*). As expected, the most influential position for gRNA sequence activity is a strong bias toward C at position 18 (Figure 4A and Table S4).

To validate the scoring scheme, we used data from TKOv1 screens that included the 85k supplemental library (HCT116 and HeLa cell lines), which added an additional six gRNAs per gene for most genes (Hart *et al.* 2015). We assigned a total score to each gRNA based on the sum of the scores at each nucleotide position in the table, where positive scores indicate a better match to the ideal sequence in the score table and negative scores indicate a worse match. We then took the subset of guides targeting core essentials and looked at the fold-change distribution in the top and bottom quartiles of scores within that subset. High scores clearly predicted better performing guides [P-value = 1.04 × 10^−38^ (HCT116) and 4.83 × 10^−50^ (HeLa), *T*-test; Figure 4, B and C] while having minimal difference on nonessential genes in both samples (data not shown). Interestingly, no sequence bias was observed in the Yusa screens (Figure 4D; P-value = 0.12). The Yusa library design already includes a sequence optimization step for the last five bases (positions 16–20, proximal to the SpCas9 PAM sequence), as well as a modified tracrRNA scaffold; these modifications were previously shown to eliminate sequence bias (Tzelepis *et al.* 2016) and our analysis is consistent with these results. However, the score table does predict somewhat improved guide performance in the Sabatini screens (Figure 4E; P-value = 2.0 × 10^−10^; Wang *et al.* 2015) and substantially better guides in the Achilles screens (Figure 4F; P-value = 1.35 × 10^−51^; Aguirre *et al.* 2016). Notably, the median gRNA score in the Sabatini library is strongly positive, implying the use of similar design rules, but the median gRNA score in GeCKOv2 is strongly negative. This observation is generally consistent with the substantially better overall performance observed in the Sabatini screens relative to the GeCKOv2 screens, as well as the increased predictive power of our gRNA sequence score for the GeCKO library.

We used the score table to design a sequence optimized CRISPR/SpCas9 library that would enable efficient screening of cell lines (see *Materials and Methods*). The TKOv3 is a one-component library (*i.e.*, *Streptococcus pyogenes* Cas9 is part of the library vector) containing 71,090 gRNAs with four gRNAs per gene, targeting a total of 18,053 protein-coding genes. The median sequence score of the gRNA in TKOv3 is 1.79, and 97.5% of gRNAs have a positive sequence score. In addition, we included 142 gRNA sequences targeting EGFP, LacZ, and luciferase for use as controls in experiments using these reporter genes.

**Figure 5:**
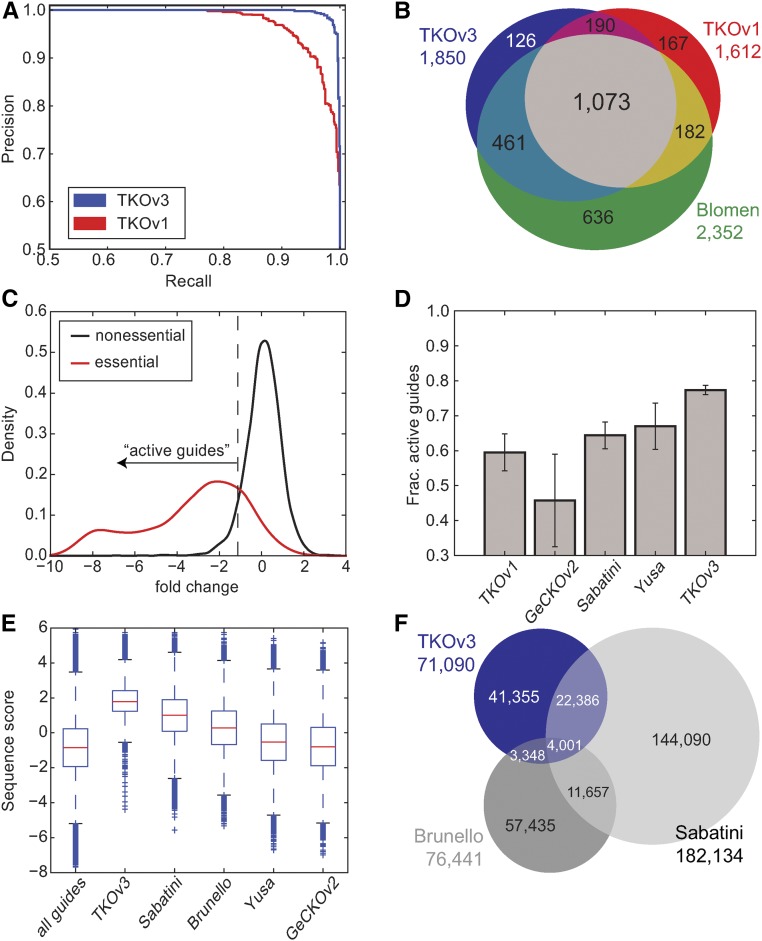
Evaluation of TKOv3 library. (A) Precision-recall curves of TKOv1 and TKOv3 screens in HAP1 cells. (B) Comparison of essential genes in TKOv3 *vs.* TKOv1 and HAP1 essentials from Blomen *et al.* (2015) at 5% FDR. (C) TKOv3 guides targeting essential *vs.* nonessential genes in HAP1. Guides targeting essential genes, with fold-change <5th percentile of guides targeting nonessential genes, are defined as active guides. (D) The fraction of active guides (active guides targeting essential genes / all guides targeting essential genes) across the five libraries tested. (E) Distribution of sequence scores for all candidate gRNA sequences (*n* ∼ 2.5 million) compared to published CRISPR/SpCas9 libraries. (F) Overlap of gRNA sequences in the top three libraries by sequence score.

The TKOv3 library was subsequently used to screen HAP1 cells and the results were compared to screens in HAP1 cells using the TKOv1 library. Importantly, both sets of screens were performed under the same experimental conditions: a single large-scale infection divided into three replicates, with genomic DNA collected after six serial passages. CEG2 and previously defined reference nonessentials (Hart *et al.* 2014) were subsequently used to train and test the BAGEL pipeline for both screens, and as shown in Figure 5A, the smaller, sequence-optimized TKOv3 library outperformed the TKOv1 library by precision-recall analysis. The TKOv3 results also recovered more essential genes at a strict threshold (BF > 6 and FDR < 3%; *n* = 1850 for TKOv3 *vs.* *n* = 1612 for TKOv1), and more of these hits intersect with a list of fitness genes identified in the same cell line, using a comprehensive gene trap screen (Blomen *et al.* 2015) [*n* = 1534 for TKOv3 *vs.* *n* = 1255 for TKOv1, out of *n* = 2352 fitness genes at 5% FDR identified in Blomen *et al.* (2015); Figure 5B].

As a further means of comparing library quality, we examined the observed dropout of guides targeting essential and nonessential genes. As expected, guides targeting reference nonessential genes showed a largely symmetric distribution of (log) fold-changes centered at zero (Figure 5C). We defined a scoring metric, the fraction of active guides, as the percentage of gRNAs targeting reference essential genes that show a fold-change greater than that of 95% of gRNAs targeting reference nonessential genes. The TKOv3 library shows a marked improvement over the TKOv1 library, as well as other latest-generation libraries for which screening data are available (Figure 5D). All of the libraries are substantially more efficient than GeCKOv2, as evaluated by data from Aguirre *et al.* (2016). This summary statistic is consistent with a precision-recall analysis of each library, performed by analyzing each screen with the BAGEL pipeline and using the same v2 reference essential (*i.e.*, CEG2) and reference nonessential gene sets.

Another library targeting human genes and designed from empirical observations is the Brunello library, described in Doench *et al.* (2016). We currently have no negative selection screen data from this library to evaluate the fraction of active guides, so as a proxy we calculated the distribution of guide sequence scores for all gRNAs in the library. Compared to all candidate gRNAs (all potential guide sequences targeting protein-coding exons with an NGG PAM and 40–75% GC content; ∼2.5 million sequences), the Brunello library has a higher sequence score than average, though not as high as either the Sabatini or TKOv3 libraries (Figure 5E). However, it should be noted that low sequence score does not necessarily imply poor overall library performance; the Yusa library has sequence scores comparable to those in the GeCKOv2 library but due to its other design considerations (*e.g.*, 3′ sequence bias and use of modified tracrRNA), it shows markedly better performance than GeCKOv2.

Given the similar sequence signatures of both TKOv3 and the Sabatini library (Figure 5E), we examined the overlap of actual gRNA sequences between the two collections. Over 26,000 of the ∼71,000 gRNA in TKOv3 are also in the Sabatini library, comprising some 37% of TKOv3 sequences. Both TKOv3 and Sabatini libraries show markedly less overlap with the Brunello library (Figure 5F).

## Discussion

Gene knockout screening in mammalian cells is transforming human functional genomics and target discovery efforts, but CRISPR technology continues to evolve rapidly. Early proof-of-concept screens using pooled-library approaches needed large numbers of gRNAs per gene to overcome the unknown sources of variation in gRNA targeting efficiency. We analyzed panels of pooled-library screens from three different research groups, each using different CRISPR libraries, to identify a set of common hits across all tested conditions. This set of 684 genes, named CEG2, is consistent across adherent and suspension cell lines and represents a broader cross-section of essential cellular processes than the CEG1 set derived from a panel of pooled-library shRNA knockdown screens. Identification of CEG2 genes will be a useful metric for evaluating the sensitivity of genome-scale knockout screens in human cell lines.

Since the collection of known essential genes offers a set of expected outcomes for screens, we leveraged this knowledge to determine the characteristics of gRNAs that maximize the discrimination of essential genes from nonessentials. We derived a sequence signature from the TKOv1 screens that predicts improved gRNA performance in TKOv1 screens as well as those using the GeCKOv2 and Sabatini libraries. We find no improvement when we apply our score to the Yusa library, which includes both sequence optimization and a modified scaffold, although we do not attempt to deconvolve the relative contribution of each. We emphasize that our findings are specific to endonuclease-competent SpCas9, with an NGG PAM targeting coding exons. We do not address chromatin state or other factors that might affect SpCas9 binding or the creation and repair of double-strand breaks in other genomic contexts. However, this approach does present a framework for library design and evaluation using other candidate CRISPR-associated endonucleases.

We also evaluated the effect of varying the number of gRNAs per gene in the library, and observed that increasing library size beyond four gRNAs per gene typically yielded a small incremental increase in the sensitivity of the screen. Based on these observations we designed a new library, TKOv3, which contains four sequence-optimized guides targeting each of 18,053 protein-coding genes. The result is a library of 71,090 gRNA sequences that is small enough to facilitate genome-scale screens in cell lines while sensitive enough to minimize false negatives in a well-designed screen. TKOv3 library is a one-component library, expressing SpCas9 from the viral vector, which relaxes the requirement to knock SpCas9 into cell lines; however, we do not have a direct comparison of one-component and two-component libraries using the same optimized sequences.

Overall, improving the accuracy and scalability of CRISPR screens offers considerable benefits for the systematic survey of context-dependent essential genes across tissue types, genetic mutational landscapes, and environmental stimuli. Further efficiencies may be gained by exploring alternative Cas proteins, or engineering existing ones, for a variety of functions: to increase nuclease effectiveness, to broaden the addressable set of cleavage sites by using alternative PAM sequences, and especially by exploiting endogenous or engineered multiplexing capabilities. Though each alternative CRISPR-associated nuclease will almost certainly require a sequence optimization survey such as this one, the consistency of the latest-generation SpCas9 libraries suggests that we are approaching a maximally efficient SpCas9 gRNA design.

## Supplementary Material

Supplemental material is available online at www.g3journal.org/lookup/suppl/doi:10.1534/g3.117.041277/-/DC1.

## References

[bib1] Aguirre A. J. Meyers R. M. Weir B. A. Vazquez F. Zhang C. Z., 2016 Genomic copy number dictates a gene-independent cell response to CRISPR-Cas9 targeting. Cancer Discov. 6: 914–929. 27260156 10.1158/2159-8290.CD-16-0154 PMC4972686

[bib2] Blomen V. A. Majek P. Jae L. T. Bigenzahn J. W. Nieuwenhuis J., 2015 Gene essentiality and synthetic lethality in haploid human cells. Science 350: 1092–1096. 26472760 10.1126/science.aac7557

[bib3] Doench J. G. Fusi N. Sullender M. Hegde M. Vaimberg E. W., 2016 Optimized sgRNA design to maximize activity and minimize off-target effects of CRISPR-Cas9. Nat. Biotechnol. 34: 184–191. 26780180 10.1038/nbt.3437 PMC4744125

[bib4] Echeverri C. J. Beachy P. A. Baum B. Boutros M. Buchholz F., 2006 Minimizing the risk of reporting false positives in large-scale RNAi screens. Nat. Methods 3: 777–779. 16990807 10.1038/nmeth1006-777

[bib5] Evers B. Jastrzebski K. Heijmans J. P. Grernrum W. Beijersbergen R. L., 2016 CRISPR knockout screening outperforms shRNA and CRISPRi in identifying essential genes. Nat. Biotechnol. 34: 631–633. 27111720 10.1038/nbt.3536

[bib6] Haeussler M. Schonig K. Eckert H. Eschstruth A. Mianne J., 2016 Evaluation of off-target and on-target scoring algorithms and integration into the guide RNA selection tool CRISPOR. Genome Biol. 17: 148. 27380939 10.1186/s13059-016-1012-2 PMC4934014

[bib7] Hart T. Moffat J., 2016 BAGEL: a computational framework for identifying essential genes from pooled library screens. BMC Bioinformatics 17: 164. 27083490 10.1186/s12859-016-1015-8 PMC4833918

[bib8] Hart T. Brown K. R. Sircoulomb F. Rottapel R. Moffat J., 2014 Measuring error rates in genomic perturbation screens: gold standards for human functional genomics. Mol. Syst. Biol. 10: 733. 24987113 10.15252/msb.20145216 PMC4299491

[bib9] Hart T. Chandrashekhar M. Aregger M. Steinhart Z. Brown K. R., 2015 High-resolution CRISPR screens reveal fitness genes and genotype-specific cancer liabilities. Cell 163: 1515–1526. 26627737 10.1016/j.cell.2015.11.015

[bib10] Heigwer F. Kerr G. Boutros M., 2014 E-CRISP: fast CRISPR target site identification. Nat. Methods 11: 122–123. 24481216 10.1038/nmeth.2812

[bib11] Heigwer F. Zhan T. Breinig M. Winter J. Brugemann D., 2016 CRISPR library designer (CLD): software for multispecies design of single guide RNA libraries. Genome Biol. 17: 55. 27013184 10.1186/s13059-016-0915-2 PMC4807595

[bib12] Hsu P. D. Scott D. A. Weinstein J. A. Ran F. A. Konermann S., 2013 DNA targeting specificity of RNA-guided Cas9 nucleases. Nat. Biotechnol. 31: 827–832. 23873081 10.1038/nbt.2647 PMC3969858

[bib13] Jinek M. Chylinski K. Fonfara I. Hauer M. Doudna J. A., 2012 A programmable dual-RNA-guided DNA endonuclease in adaptive bacterial immunity. Science 337: 816–821. 22745249 10.1126/science.1225829 PMC6286148

[bib14] Kaelin W. G. Jr, 2012 Molecular biology. Use and abuse of RNAi to study mammalian gene function. Science 337: 421–422. 22837515 10.1126/science.1225787 PMC3705935

[bib15] Koike-Yusa H. Li Y. Tan E. P. Velasco-Herrera Mdel C. Yusa K., 2014 Genome-wide recessive genetic screening in mammalian cells with a lentiviral CRISPR-guide RNA library. Nat. Biotechnol. 32: 267–273. 24535568 10.1038/nbt.2800

[bib16] Liu X. Homma A. Sayadi J. Yang S. Ohashi J., 2016 Sequence features associated with the cleavage efficiency of CRISPR/Cas9 system. Sci. Rep. 6: 19675. 26813419 10.1038/srep19675 PMC4728555

[bib17] Munoz D. M. Cassiani P. J. Li L. Billy E. Korn J. M., 2016 CRISPR screens provide a comprehensive assessment of cancer vulnerabilities but generate false-positive hits for highly amplified genomic regions. Cancer Discov. 6: 900–913. 27260157 10.1158/2159-8290.CD-16-0178

[bib18] Park J. Kim J. S. Bae S., 2016 Cas-Database: web-based genome-wide guide RNA library design for gene knockout screens using CRISPR-Cas9. Bioinformatics 32: 2017–2023. 27153724 10.1093/bioinformatics/btw103 PMC4920116

[bib19] Parnas O. Jovanovic M. Eisenhaure T. M. Herbst R. H. Dixit A., 2015 A genome-wide CRISPR screen in primary immune cells to dissect regulatory networks. Cell 162: 675–686. 26189680 10.1016/j.cell.2015.06.059 PMC4522370

[bib20] Shalem O. Sanjana N. E. Hartenian E. Shi X. Scott D. A., 2014 Genome-scale CRISPR-Cas9 knockout screening in human cells. Science 343: 84–87. 24336571 10.1126/science.1247005 PMC4089965

[bib21] Steinhart Z. Pavlovic Z. Chandrashekhar M. Hart T. Wang X., 2017 Genome-wide CRISPR screens reveal a Wnt-FZD5 signaling circuit as a druggable vulnerability of RNF43-mutant pancreatic tumors. Nat. Med. 23: 60–68. 27869803 10.1038/nm.4219

[bib22] Tzelepis K. Koike-Yusa H. De Braekeleer E. Li Y. Metzakopian E., 2016 A CRISPR Dropout screen identifies genetic vulnerabilities and therapeutic targets in acute Myeloid Leukemia. Cell Rep. 17: 1193–1205. 27760321 10.1016/j.celrep.2016.09.079 PMC5081405

[bib23] Wang T. Wei J. J. Sabatini D. M. Lander E. S., 2014 Genetic screens in human cells using the CRISPR-Cas9 system. Science 343: 80–84. 24336569 10.1126/science.1246981 PMC3972032

[bib24] Wang T. Birsoy K. Hughes N. W. Krupczak K. M. Post Y., 2015 Identification and characterization of essential genes in the human genome. Science 350: 1096–1101. 26472758 10.1126/science.aac7041 PMC4662922

[bib25] Wang T. Yu H. Hughes N. W. Liu B. Kendirli A., 2017 Gene essentiality profiling reveals gene networks and synthetic lethal interactions with Oncogenic Ras. Cell 168: 890–903 e15. 28162770 10.1016/j.cell.2017.01.013 PMC5445660

